# Ubiquitination-Induced Fluorescence Complementation (UiFC) for Detection of K48 Ubiquitin Chains *In Vitro* and in Live Cells

**DOI:** 10.1371/journal.pone.0073482

**Published:** 2013-09-05

**Authors:** Zhiliang Chen, Yongwang Zhong, Yang Wang, Shan Xu, Zheng Liu, Ilia V. Baskakov, Mervyn J. Monteiro, Mariusz Karbowski, Yuxian Shen, Shengyun Fang

**Affiliations:** 1 Center for Biomedical Engineering and Technology, Anhui Medical University, Hefei, China; 2 Department of Physiology, Anhui Medical University, Hefei, China; 3 School of Basic Medical Science and Biopharmaceutical Research Institute, Anhui Medical University, Hefei, China; 4 Department of Biochemistry and Molecular Biology, University of Maryland, Baltimore, Maryland, United States of America; Karolinska Institutet, Sweden

## Abstract

Proteins can be modified with eight homogenous ubiquitin chains linked by an isopeptide bond between the C-terminus of one ubiquitin and an amine from one of the seven lysines or the N-terminal methionine of the next ubiquitin. These topologically distinct ubiquitin chains signal for many essential cellular functions, such as protein degradation, cell cycle progression, DNA repair, and signal transduction. The lysine 48 (K48)-linked ubiquitin chain is one of the most abundant chains and a major proteasome-targeting signal in cells. Despite recent advancements in imaging linkage-specific polyubiquitin chains, no tool is available for imaging K48 chains in live cells. Here we report on a ubiquitination-induced fluorescence complementation (UiFC) assay for detecting K48 ubiquitin chains *in vitro* and in live cells. For this assay, two nonfluorescent fragments of a fluorescent protein were fused to the ubiquitin-interacting motifs (UIMs) of epsin1 protein. Upon simultaneous binding to a ubiquitin chain, the nonfluorescent fragments of the two fusion proteins are brought in close proximity to reconstitute fluorescence. When used *in vitro*, UiFC preferentially detected K48 ubiquitin chains with excellent signal-to-noise ratio. Time-lapse imaging revealed that UiFC is capable of monitoring increases in polyubiquitination induced by treatment with proteasome inhibitor, by agents that induce stress, and during mitophagy in live cells.

## Introduction

Ubiquitin is a 76 amino acids long protein that is highly conserved in all eukaryotes. It can be conjugated to proteins as either a single moiety or in a form of ubiquitin chain. The chain of ubiquitin is built by formation of an isopeptide bond between the C-terminus of one ubiquitin and an amine from one of its seven internal lysine residues (K6, K11, K27, K29, K33, K48, K63) or the amino terminal methionine (Met-1) of the next ubiquitin [1–3]. Cells also make free ubiquitin chains that do not attach to any protein [4,5]. Moreover, ubiquitin chains can be formed through mixed linkages and even with ubiquitin-like proteins, such as the small ubiquitin-like modifier (SUMO) [6]. To complicate matters further, cells use deubiquitinating enzymes (DUBs) to disassemble ubiquitin chains [2,7]. These topologically distinct ubiquitin chains and their disassembly help govern remarkably functional diversity in ubiquitin signaling, such as targeting proteins for degradation, apoptosis, signal transduction, gene transcription, DNA repair, cell cycle progression, immune responses, virus budding, protein trafficking, and receptor and channel endocytosis [8,9]. Many of these functions control the life and death of cells. Accordingly, aberrant ubiquitination has been widely associated with development of a wide range of devastating diseases, such as malignancies, inflammatory disorders, and neurodegenerative diseases [10].

Signaling through ubiquitin chains is achieved by a number of proteins containing ubiquitin-binding domains (UBDs), such as the ubiquitin-interacting motif (UIM) and ubiquitin-associated (UBA) domains [9,11]. These proteins interact with both ubiquitin chains, often in a linkage-specific fashion, and the downstream effector proteins of signaling pathways. Through linkage-specific interactions, UBD-containing proteins decode ubiquitin chain-dependent signals to the desired biological function. For example, numerous E3 ubiquitin ligases can assemble K48 chain on their substrate proteins. Shuttling factors, such as yeast Rad23, Dsk2 and Ddi1 or its metazoan orthologs can bind to the conjugated-K48 chain through their UBA domains and deliver the substrates to the proteasomes for degradation [12–16]. During DNA damage response (DDR), K63 chains are synthesized at the damage site by RNF8-Ubc13 complex [17–19]. These K63 chains are subsequently recognized by UIMs of receptor-associated protein 80 (Rap80), which facilitates the recruitment of several DDR mediators, including the BRCA1/BARD1 E3 ubiquitin ligase complex to the damage sites [17–19]. The transforming growth factor-β-activated kinase 1 (TAK1) plays an essential role in TNFα-mediated NFκB nuclear translocation [20]. TAK1 activation requires binding of K63 chain specifically by the Npl4 zinc finger (NZF) of its subunits Tak1-binding protein 2 and 3 (TAB 2 and TAB 3) [20,21]. The linear ubiquitin chain assembly complex (LUBAC) E3 assembles linear Met-1 ubiquitin chain that serves as signal for proteins containing ubiquitin-binding in ABIN and NEMO (UBAN) domain, such as NEMO, ABIN1-3, and optineurin, that regulate NFκB activation or autophagic elimination of bacteria [22,23]. From these studies it is clear that cells utilize a highly complex and diverse repertoire of ubiquitin chains for different purposes. Much more work remains to be done to understand the decoding process of chain linkage-specific functions.

An important aspect in understanding ubiquitin-chain function is the ability to analyze linkage-specific ubiquitination. Techniques that have been used for such analysis include mass spectrometry [24–26], lysine residue mutant ubiquitin constructs or linkage-specific monoclonal antibodies, in combination with *in vitro* ubiquitination, immunoblotting and/or fluorescent microscopy [27–30]. Ubiquitin-mediated fluorescence complementation (UbFC) in combination with expression of lysine residue mutant ubiquitin was the first technique used to determine linkage-specific ubiquitination in live cells [31]. Another more recent technique employed linkage-specific UBD tagged with a fluorescent protein to report on the localization of linear K63 and Met-1 chains in live cells [23,32]. Because the UBD of these sensors competes with the endogenous K63 or Met-1 ubiquitin-binding decoders, they have also been used to inhibit the functions mediated by these chains including TNF and IL1-induced NFκB activation [23,32].

In this study, we use a novel ubiquitination-induced fluorescence complementation (UiFC) strategy to detect ubiquitin chains in live cells. We show that UiFC preferentially detects K48 chains *in vitro* with excellent signal response and reproducibility. Time-lapse imaging revealed that UiFC is capable of monitoring increases in K48 polyubiquitination in live cells, such as that seen after proteasome inhibition, or after treatment with agents that cause stress and during Parkin-dependent mitophagy.

## Results

### UiFC preferentially detects K48 chains in vitro

**Figure 1:**
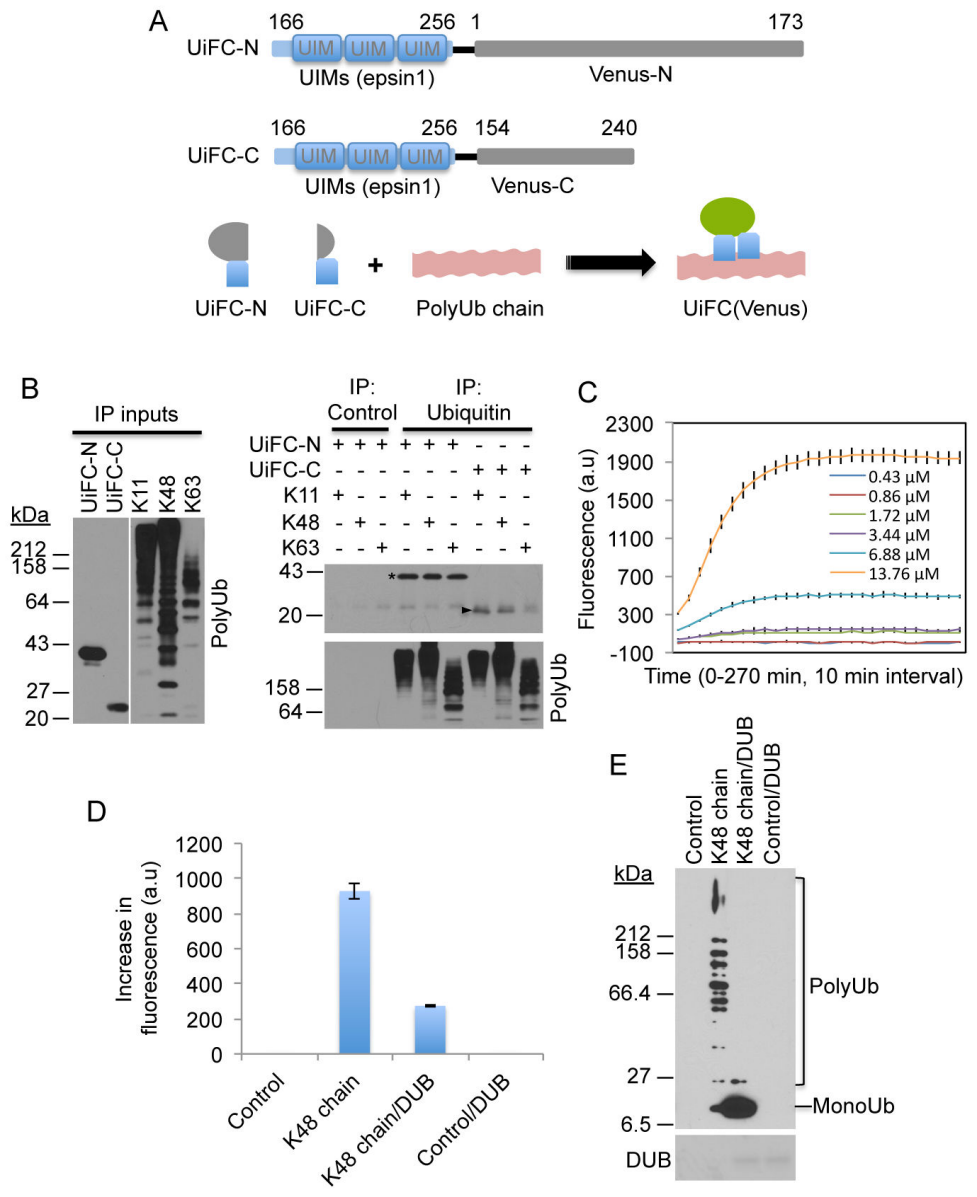
Design of ubiquitination-induced fluorescence complementation (UiFC) assay. (A) Schematic representation of UiFC-N and UiFC-C and the proposed mechanism of UiFC for detecting polyubiquitin chain. (B) UiFC-N and UiFC-C interact with K11, K48, and K63 chains. Purified 6His-UiFC-N and 6His-UiFC-C were incubated with K11, K48, or K63 chains. Following the incubation immunoprecipitation was carried out with anti-ubiquitin antibody followed by immunoblotting for venus. Control IP: anti-HA antibody was used instead of anti-ubiquitin. Input: 10% of total UiFC-N and UiFC-C or polyubiquitin chains. Aristerisk indicates UiFC-N. Arrowhead indicates UiFC-C. (C) Spontaneous reconstitution of the N and C-terminal fragment of venus in UiFC-N and UiFC-C *in vitro*. Increasing amounts of 6His-UiFC-N and 6His-UiFC-C were incubated in 20 µl Tris buffer and venus fluorescence was measured every 10 minutes for up to 270 minutes at 37 °C as described in Experimental Procedures. The mean +/- SD of venus fluorescence intensities from triplicate reactions at each time point was plotted. (D) K48 ubiquitin chain but not mono-ubiquitin induces increases in venus fluorescence. UiFC-N and UiFC-C (0.86 µM) were incubated with the reaction mix for K48 chain synthesis containing 2 µM ubiquitin in the presence or absence of ATP for 60 minutes. Control: no K48 chains, DUB: 6His-EBV-DUB. The graph shows the mean +/- SD of UiFC fluorescence intensities from triplicate reactions (left). (E) The reaction mixes used in D were analyzed by immunoblotting for ubiquitin after the measurement of venus fluorescence. Lower panel: the added 6His-EBV-DUB revealed by Ponceau staining.

Bimolecular fluorescence complementation (BiFC) has been widely used for studying protein–protein interactions in live cells [33]. It is based on reconstitution of fluorescence of two nonfluorescent fragments of a fluorescent protein when they are brought in close proximity to each other by an interaction between proteins fused to the fragments. We investigated whether BiFC could be modified to monitor changes in ubiquitin chains by using UBD fused with nonfluorescent fragments of a fluorescent protein as the sensors. We anticipated that multiple UBDs would bind to a polyubiquitin chain simultaneously. This simultaneous binding would bring the two nonfluorescent fragments (fused to the UBD) in close proximity leading to complementation of fluorescence (Figure 1A). To test this possibility, we selected the three tandem UIMs from epsin1 protein since *in vitro* pull-down assays demonstrated that these UIMs in tandem bind K48 and K63 chains but not monomeric ubiquitin [34]. We selected venus, a variant of yellow fluorescent protein that has been widely used for BiFC [35]. Accordingly, we generated plasmid constructs for expressing the three tandem UIMs (referred to as UIM hereafter) fused with the N-terminal (amino acid residues 1-173) or C-terminal (amino acid residues 154-240) nonfluorescent fragments of venus protein, called UiFC-N and UiFC-C (Figure 1A). A 14 amino acid linker rich in glycine was fused at the C-terminus of the UIMs to increase the flexibility of the attached venus fragments. We first determined whether the UIM fused to the N- and C-terminal portions of venus retained their ability to interact with polyubiquitin chains. K11, K48 and K63 polyubiquitin chains were synthesized *in vitro* using methods reported previously [36–38]. These ubiquitin chains were incubated with recombinant 6His-tagged UiFC-N or UiFC-C and then immunoprecipitated with anti-ubiquitin antibody. Immunoblotting of the precipitates revealed that both UiFC-N and UiFC-C are capable of interaction with all three types of ubiquitin chains (Figure 1B).

Previous studies have shown that the N- and C-terminal fragments of venus exhibits high fluorescence intensity when used in BiFC, but can re-associate spontaneously [35,39,40]. We therefore assessed the degree to which the UiFC-N and UiFC-C re-associate *in vitro*. Increasing amounts of 6His-UiFC-N and 6His-UiFC-C were co-incubated and venus fluorescence was monitored on TECAN Infinite 200Pro fluorescence microplate reader. We found that incubation of 6His-UiFC-N and 6His-UiFC-C at concentrations above 1.72 µM exhibited a dose-dependent increase in venus fluorescence that reached a plateau in 2 hours (Figure 1C), indicating that the spontaneous association is UiFC-N and UiFC-C concentration-dependent. Next, we determined whether UiFC-N and UiFC-C could detect K48 ubiquitin chains *in vitro*. To minimize background fluorescence generated from spontaneous association of the UiFC-N and UiFC-C, the assays were conducted using 0.86 µM of the proteins, which is below the 1.72 µM threshold level we found was required for inducing spontaneous re-association. The results showed that the reaction containing K48 chains generated significant amount of venus fluorescence (Figure 1D), whereas a similar reaction to which we added highly active deubiquitinating enzyme from Epstein–Barr virus (EBV-DUB) [41] to disassemble ubiquitin chains had significantly decreased fluorescence (Figure 1D). Disassembly of ubiquitin chains by EBV-DUB was confirmed by immunoblotting for ubiquitin (Figure 1E). A separate assay substituting mono-ubiquitin for K48 chains produced no fluorescence (Figure 1D, E). These results suggest that simultaneous binding of UiFC-N and UiFC-C to K48 chains induces fluorescence complementation *in vitro*.

**Figure 2:**
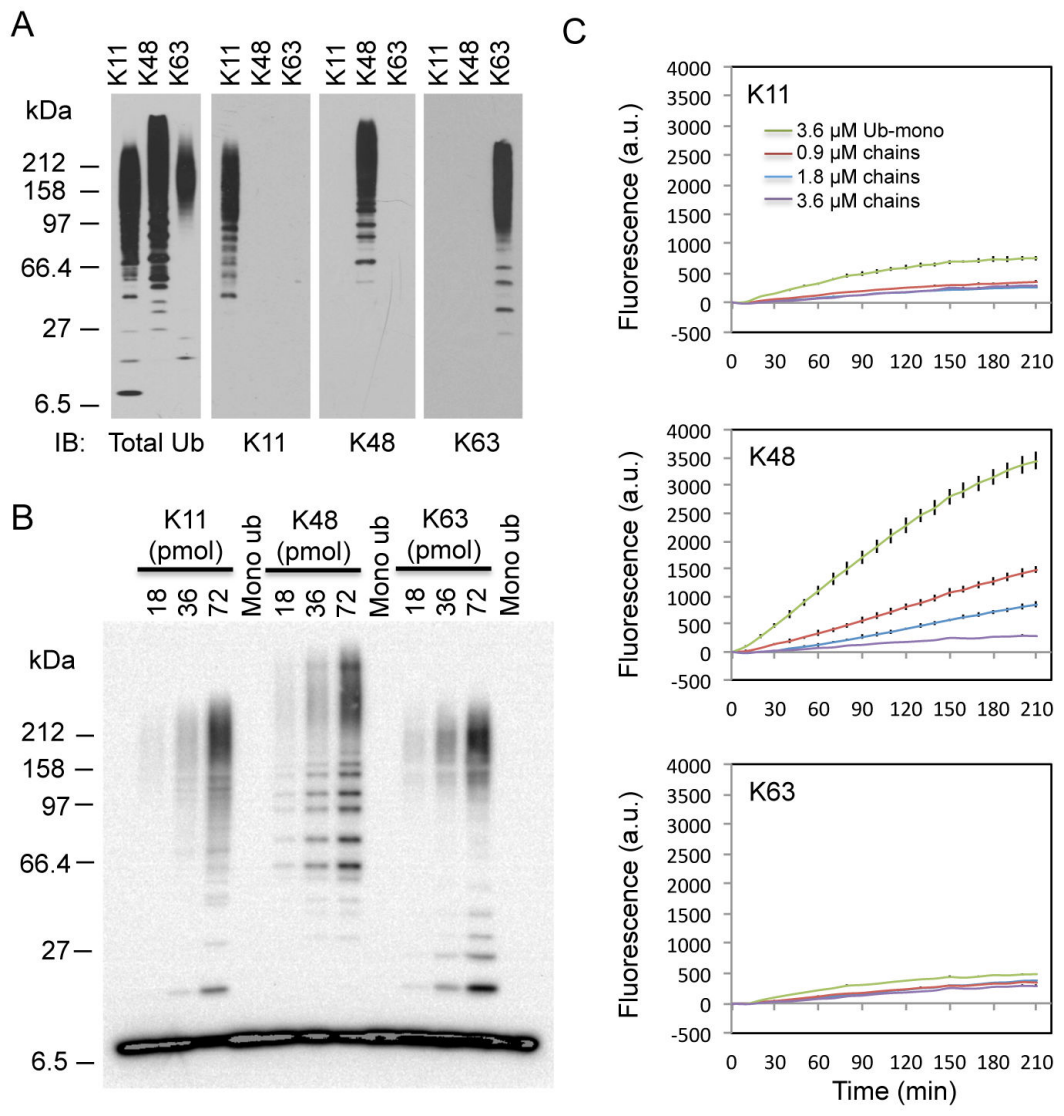
UiFC-N and UiFC-C preferentially detects with K48 ubiquitin chains *in vitro*. (A) Characterization of polyubiquitin chains with K11, K48, or K63 linkage. Ubiquitin chains were assembled *in vitro* (see Experimental Procedures for details) and analyzed by immunoblotting with linkage-specific antibody. The blots were from the same membrane stripped and re-blotted with antibody against total ubiquitin, K11 chain, K48 chain, and K63 chain, respectively. (B) Increasing amounts of K11, K48, or K63 chains (formed by18, 36, and 72 pmols of mono-ubiquitin) and mono-ubiquitin (72 pmols) were analyzed by immunoblotting for ubiquitin. Mono-ubiquitin was added to make the total amount of ubiquitin equal to 72 pmols in all lanes. (C) Measurement of UiFC fluorescence in reactions containing increasing amounts of ubiquitin chains, 6His-UiFC-N, and 6-His-UiFC-C at 10 minutes interval for up to 210 minutes. Mono-ubiquitin was added to make the final concentration of ubiquitin (mono or in chains) equal to 3.6 µM in all reactions. The mean +/- SD of the UiFC fluorescence intensities from triplicate reactions at each time point was plotted.

**Figure 3:**
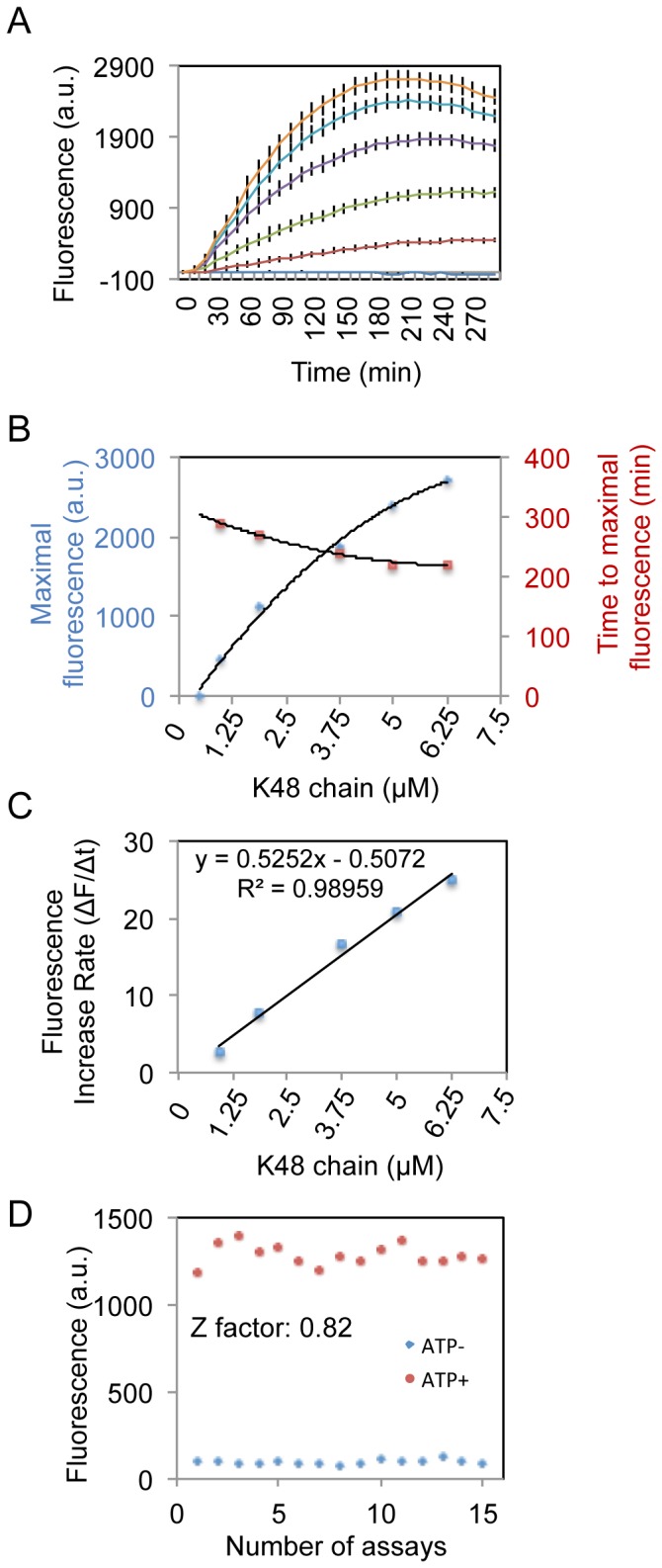
Characterization of UiFC assay *in vitro*. (A) Generation of UiFC fluorescence is time and K48 chain concentration-dependent. Increasing concentrations of K48 chains were incubated with 6His-UiFC-N and 6His-UiFC-C and the UiFC fluorescence was measured during incubation at 10 minutes interval for 280 minutes. The mean +/- SD of the UiFC fluorescence intensities from triplicate reactions at each time point was plotted. The lines from bottom to top represent 0.45, 0.9, 1.8, 3.6, 5, and 6 µM ubiquitin chains, respectively. (B) The maximal UiFC fluorescence intensity correlates well with K48 chain concentration but time required to achieve maximal fluorescence is much less dependent on ubiquitin chain concentration. (C) Kinetics of UiFC fluorescence in the detection of K48 chain. Time-dependent increases in fluorescence (ΔF/Δt) in linear region shown in A are plotted against K48 chain concentration. (D) Z factor of UiFC. 15 UiFC assays were performed and Z factor is calculated as described in Experimental Procedure. An example is shown in the graph.

K48, K63 and K11 are the most predominant polyubiquitin chains in cells [26]. We confirmed that the UIMs of epsin1 interact with K48 and K63 chains [34] and further showed that the UIMs also bind K11 chains (Figure 1B). To determine whether UiFC has ubiquitin chain linkage specificity, we incubated UiFC-N and UiFC-C with equal amount of K11, K48 or K63 chains. The chain linkage was further confirmed by immunoblotting with linkage-specific antibodies (Figure 2A). Surprisingly, UiFC preferentially detected K48 chains over K11 and K63 chains (Figure 2B, C), suggesting that the conformation of K48 but not K11 and K63 chains favors reconstitution of the venus fluorescence. Further characterization showed that the sensitivity of UiFC for detection of K48 chains is 0.9 µM (mono-ubiquitin concentration used for synthesis of the chains) (Figure 3A). The maximal fluorescence intensity and the kinetics of fluorescence changes (ΔF/Δt, F = fluorescence intensities) are K48 chain concentration-dependent (Figure 3B, C). In addition, the time required for achieving the maximal fluorescence exhibited a weak K48 chain-concentration dependence, suggesting that the reconstitution of venus fluorescence from the N- and C-terminal fragments is the rate-limiting step for UiFC. To determine the signal response and reproducibility of UiFC, we assessed the Z factor of UiFC and obtained a score from 0.82 to 0.92 (an example is given in Figure 3D). Since the Z factor for an “excellent” to “ideal” assay is from 0.5 to 1.0 [42], respectively, the results indicate that UiFC has excellent signal response and reproducibility *in vitro*.

### UiFC in live cells

**Figure 4:**
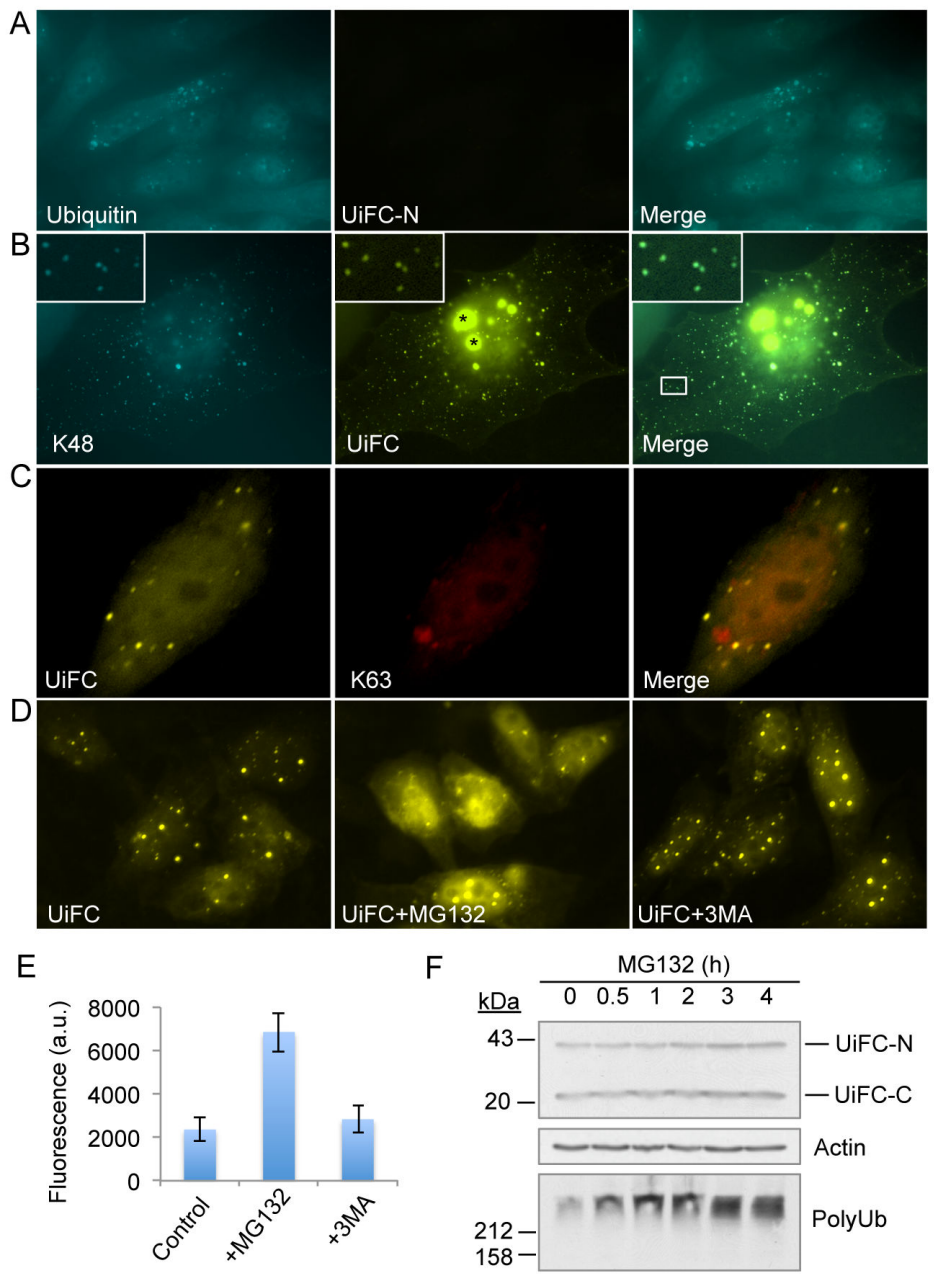
UiFC fluorescence colocalize well with K48 chains in cells. (A) Expression of UiFC-N alone does not produce any venus fluorescence in cells. HeLa cells were transfected with plasmid encoding UiFC-N and stained with anti-ubiquitin antibody (FK2) (left panel). (B) Co-localization of UiFC fluorescence (yellow) with anti-K48 chain immunofluorescence (blue, pseudo-colored to cyan to increase visibility) except for the large intranuclear UiFC aggregates (asterisk, see text for explanation). UiFC-N and UiFC-C co-transfected cells were stained with anti-K48 chain antibody. Inset: co-localization of UiFC puncta and K48 chains. (C) UiFC fluorescence (yellow) and K63 chains (red) are largely localized in different subcellular structures. Plasmids encoding UiFC-N, UiFC-C, and mCherry-tagged K63 chain sensor were transfected into HeLa cells. (D) Inhibition of proteasome but not autophagy pathway increases UiFC fluorescence. Co-expression of UiFC-N and UiFC-C produces diffused fluorescence and fluorescent puncta (yellow) throughout the cell (left panel); Treatment with MG132 (10 µM, middle panel) but not 3-MA (20 mM, right panel) for 4 hours leads to increases in UiFC fluorescence. UiFC: UiFC-N and UiFC-C. (E) Quantification of UiFC fluorescence intensities in control cells and cells treated with MG132 or 3MA. The fluorescence intensity of individual cell was measured using ImageJ software. The graph shows the mean +/- SD of UiFC fluorescence, *n* ≥ 53. (F) Effects of proteasome inhibition on UiFC-N and UiFC-C levels. HeLa cells co-transfected with UiFC-N and UiFC-C were treated with MG132 (10 µM) for the indicated time and the levels of UiFC-N and UiFC-C were determined by immunoblotting. Polyubiquitin was blotted to show the efficiency of MG132. Actin was blotted as a loading control.

We next determined whether UiFC is suitable for detecting ubiquitin chains in live cells. We first expressed the UiFC-N alone in HeLa cells as a negative control and, as expected, found no reconstitution of venus fluorescence (Figure 4A, middle panel) despite clear evidence that the cells contained anti-ubiquitin antibody staining (Figure 4A, left panel). However, when UiFC-N and UiFC-C were co-expressed, venus fluorescence was reconstituted and appeared as many puncta diffusely throughout the cytoplasm and nucleus (Figure 4B, left panel). Interestingly, the venus fluorescent puncta colocalized well with anti-K48 chain antibody staining, with the exception of the very large intranuclear aggregates (Figure 4B, asterisks in middle panel). To determine whether K63 chains were also present in the UiFC-positive puncta, we co-expressed UiFC-N and UiFC-C along with the recently developed K63 chain sensor tagged with mCherry [32], and found that their fluorescence patterns did not significantly overlap (Figure 4C). This result suggests this UIM-based UiFC preferentially detects K48 chains over K63 chains in cells.

Because K48-linked chains target proteins for proteasomal degradation, we next examined whether inhibition of proteasome activity altered UiFC fluorescence. Indeed, treatment of cells co-expressing UiFC-N and UiFC-C with proteasome inhibitor MG132 but not autophagy inhibitor 3-Methyladenine (3MA) significantly increased venus fluorescence (Figure 4D, middle panel vs. left and right panels, and E). To determine whether UiFC-N and UiFC-C are themselves proteasomal substrates, we treated cells expressing these two fusion proteins with MG132 for up to 4 hours. Immunoblotting revealed that the treatment caused slightly increases in the levels of UiFC proteins, but induced robust buildup of polyubiquitin chains (Figure 4F), indicating that UiFC-N and UiFC-C proteins are not inherently unstable. Thus, the large increase in venus fluorescence seen after MG132-treatment is unlikely to stem from the small decrease in the stability of the fusion proteins for UiFC, rather due to the accumulation of polyubiquitinated proteasomal substrates.

**Figure 5:**
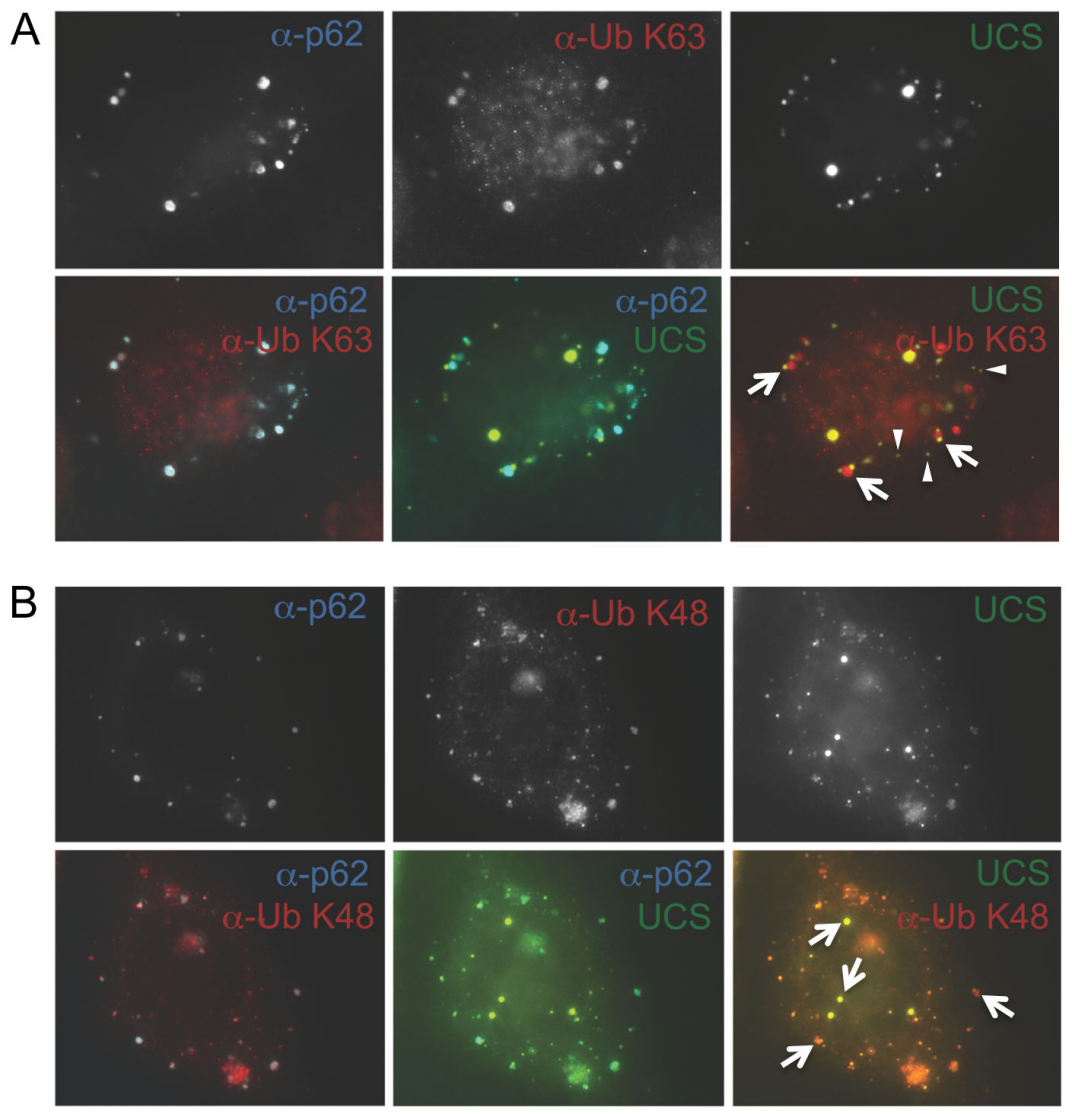
UiFC fluorescence colocalize well with K48 chains but not K63 chains enriched in p62/SQSTM1 bodies. K63 chains colocalize well with p62/SQSTM1 bodies (lower left) but not with UiFC fluorescence (lower right). HeLa cells transfected with plasmids encoding HA-p62/SQSTM1 and UiFC were processed for immunostaining with mouse monoclonal anti-p62/SQSTM1 (blue) and rabbit monoclonal anti-K63 chain antibody (red) (A) or affinity-purified rabbit polyclonal anti-p62/SQSTM1 (blue, pseudo-colored to cyan) and mouse monoclonal anti-K48 chain antibody (red) (B), followed by fluorescent microscopy.

To further determine whether UiFC can discriminate K48 from K63 chains, we turned to p62/SQSTM1 protein. Previous studies have reported that p62/SQSTM1 contains a UBA domain that preferentially binds K63-polyubiquitinated proteins and sequesters K63 chains into p62/SQSTM1 bodies [43,44]. To increase sequestration of K63 polyubiquitinated proteins, we overexpressed p62/SQSTM1 along with UiFC-N and UiFC-C in HeLa cells and then double stained the cells with antibodies for p62/SQSTM1 and K63 or K48 chains. Consistent with the previous report, p62/SQSTM1 bodies colocalized well with K63 chains (Figure 5A, lower left panel), but much less so with K48 chains [43,44] (Figure 5B, lower left panel). Interestingly, a subset of the UiFC fluorescent puncta were found in close proximity, but did not colocalize with p62/SQSTM1 bodies (Figure 5A, arrows in lower right panel, arrowheads indicate UiFC singly labeled puncta). On the other hand, the UiFC-induced venus fluorescence colocalized well with K48 chains (Figure 5B, arrows in lower right panel). These results suggest that our UiFC reporter does not detect K63 chains in cells even under conditions where K63 chains are concentrated in p62/SQSTM1 bodies.

**Figure 6:**
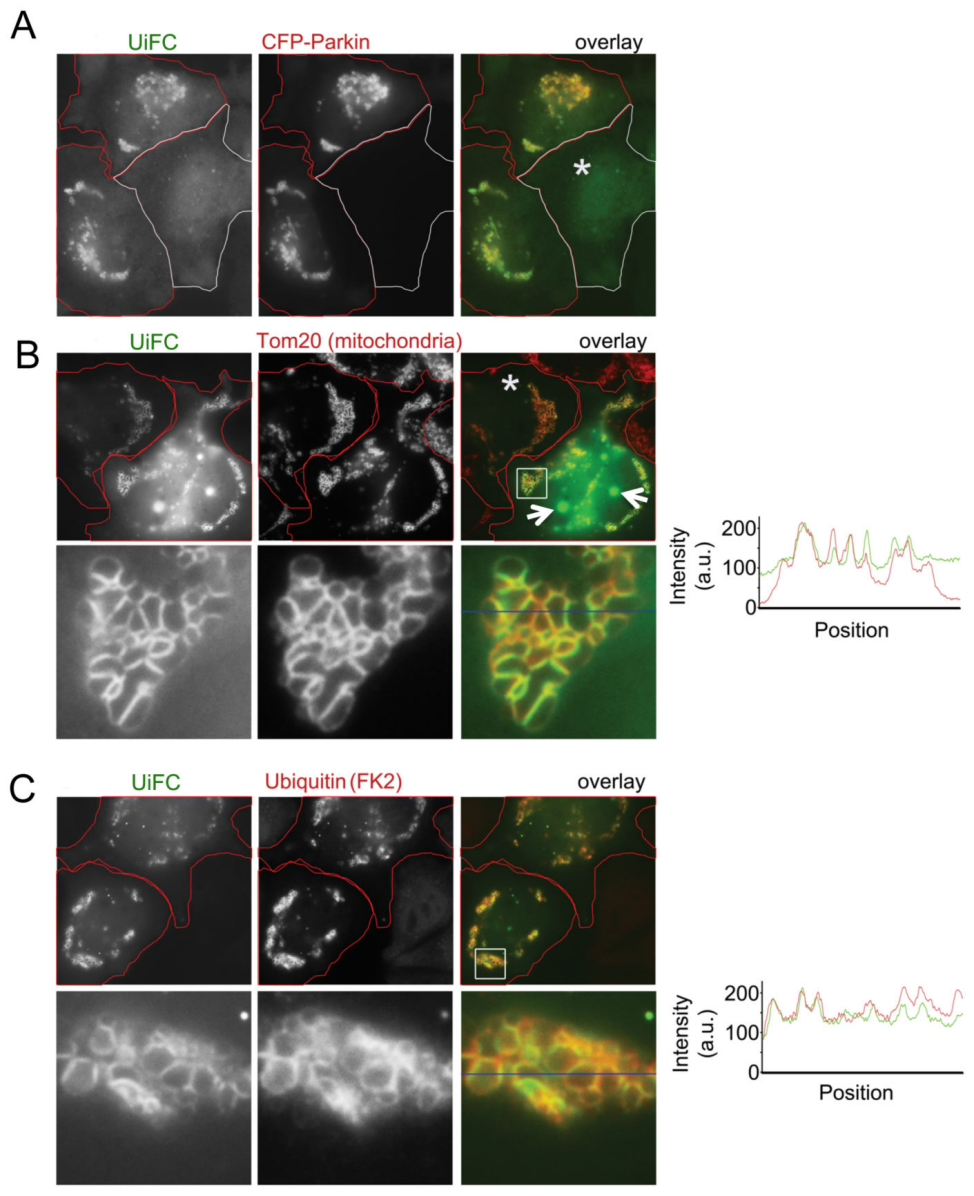
The mitochondrial outer membrane localization of UiFC in Parkin-expressing and FCCP-treated cells. (A) Parkin-dependent UiFC fluorescence associated with the mitochondria. HeLa cells co-expressing UiFC-N, UiFC-C and CFP-Parkin were treated with the uncoupler FCCP to induce mitochondrial damage and trigger mitochondrial translocation of Parkin. FCCP induced UiFC fluorescence associated with mitochondria in two cells expressing CFP-Parkin. No perinuclear UiFC aggregates seen in the cell that does not express CFP-Parkin (*). (B, C) Colocalization of UiFC and Tom20 or substrate-conjugated ubiquitin. HeLa cells co-expressing UiFC-N, UiFC-C and untagged Parkin were treated with FCCP. Cells were then processed for immunostaining for the marker of the outer mitochondrial membrane Tom20 (B), or with FK2 antibody that recognize substrate-conjugated ubiquitin (C) and analyzed by fluorescence microscopy. Tom20 and FK2 signals are red; UiFC is yellow on overlay images. The cell expressing higher level (arrows in B) but not the lower level (*) of UiFC exhibits intranuclear UiFC aggregates. The colocalization of Tom20 or ubiquitin (red) and UiFC (yellow) was also shown by the profiles of the fluorescence along blue lines in the enlarged images.

Next, we tested whether UiFC could be used for imaging and quantifying ubiquitination mediated by Parkin, an E3 ubiquitin ligase that translocates to mitochondria to ubiquitinate mitochondrial outer membrane proteins at the early stage of mitochondria-specific autophagy (mitophagy) [23,32,45]. First, we determined whether UiFC occurs during Parkin-dependent mitophagy. We co-transfected plasmids encoding CFP-Parkin, UiFC-N and UiFC-C in HeLa cells. The following day cells were treated with carbonyl cyanide p-(trifluoromethoxy) phenylhydrazone (FCCP), an uncoupler of mitochondrial oxidative phosphorylation, to induce mitochondrial damage and subsequent CFP-Parkin translocation to the mitochondria. The results showed that UiFC fluorescence colocalized well with CFP-Parkin clusters (Figure 6A). CFP-Parkin-expressing cells without FCCP treatment and cells that did not express CFP-Parkin did not show UiFC fluorescence clusters (Figure 6A, asterisk). To determine whether UiFC fluorescence decorated damaged mitochondria, HeLa cells co-expressing UiFC-N, UiFC-C and untagged Parkin were treated with FCCP followed by staining for Tom20 as an mitochondrial marker and substrate-conjugated ubiquitin. UiFC fluorescence colocalized well with Tom20 and conjugated ubiquitin stained by FK2 antibody (Figure 6B, C). These results suggest that during FCCP-induced mitophagy, UiFC detects ubiquitination of outer mitochondrial membrane proteins mediated by Parkin.

**Figure 7:**
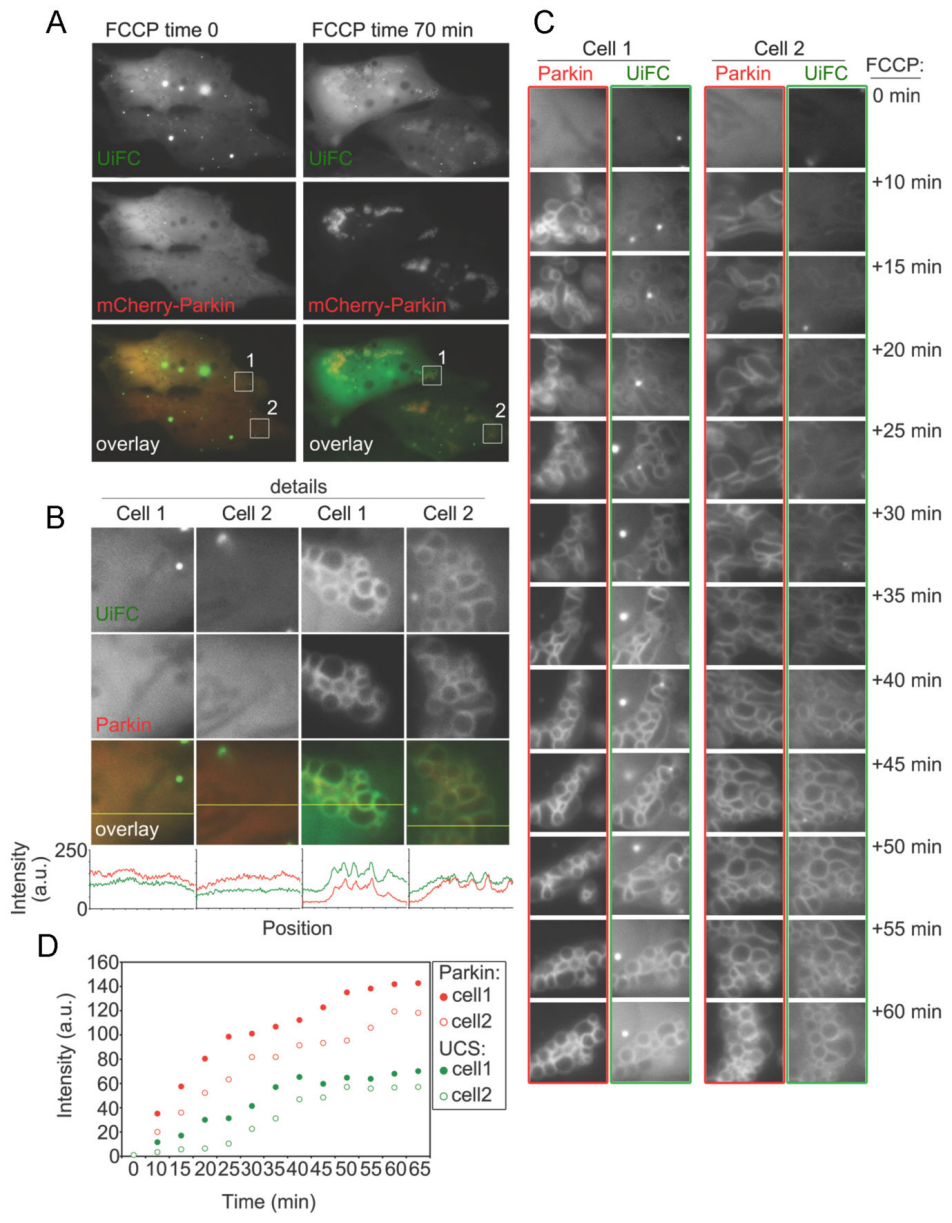
Application of UiFC to image Parkin-dependent ubiquitination of mitochondrial outer membrane proteins. (A) Cells co-expressing UiFC-N, UiFC-C (yellow on overlay images) and mCherry-Parkin (red on overlay images) were treated with the uncoupler FCCP to induce mitochondrial damage and mitochondrial translocation of mCherry-Parkin. An example of the time-lapse studies is shown. In (A) the same cells at time 0 and 70 min after addition of FCCP are depicted. In (B) details from boxed areas in (A), as well as plot profiles along the yellow lines in overlay detail images are shown (red is mCherry-Parkin, yellow UiFC). (C) Detailed time-lapse analyzes of the changes in mCherry-Parkin and UiFC over the time of FCCP-treatment in cells shown in (A) are depicted. Changes in the mCherry-Parkin and the UiFC fluorescence intensities normalized to the cytosolic background fluorescence intensities were also quantified and plotted as a function of treatment time (D).

To obtain temporal information on the translocation of Parkin to mitochondria and generation of UiFC fluorescence, we performed time-lapse imaging. HeLa cells were co-transfected with plasmids encoding UiFC-N, UiFC-C and mCherry-Parkin. Imaging of mCherry-Parkin and UiFC fluorescence began immediately after addition of FCCP at 5-minute interval for up to 70 minutes. After 70-minute treatment with FCCP, mCherry-Parkin and UiFC fluorescence became clustered and well colocalized (Figure 7A, B). Detailed analysis of the movies revealed mCherry-Parkin translocated to mitochondria first, followed by a gradual increase in UiFC fluorescence (Figure 7, Movie S1a–1c). Moreover, the intensities of UiFC fluorescence correlated well with the levels of mCherry-Parkin expression (Figure 7C, D: Cell 1 vs. Cell 2). The cells expressing more mCherry-Parkin showed a faster increase in UiFC fluorescence (Figure 7C, D). Taken together, these results indicate that our UiFC assay can be used as a tool for real-time imaging and quantification of polyubiquitination during mitophagy in live cells.

**Figure 8:**
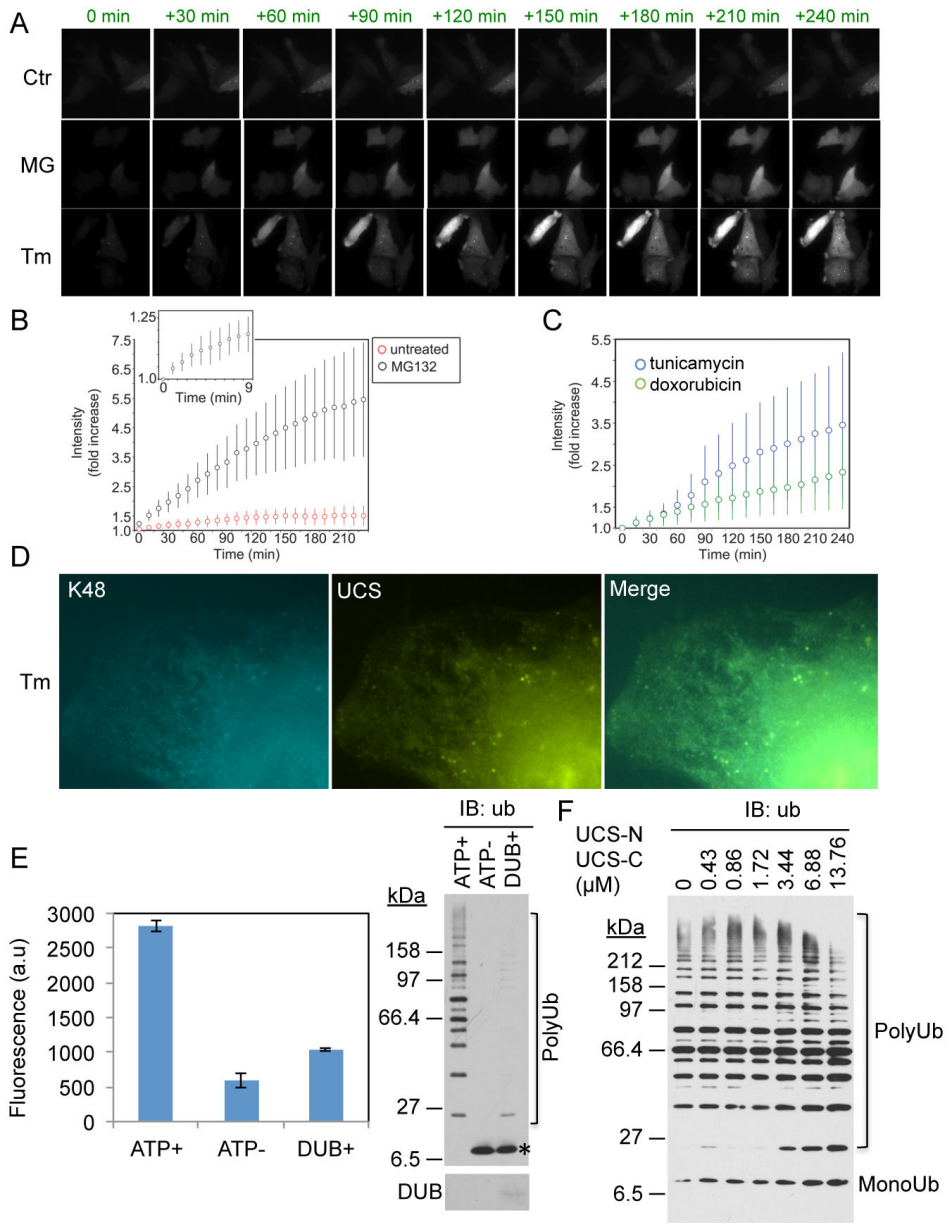
UiFC in live cells under stress. (A) Examples of time-lapse experiments of untreated (Ctr: control), proteasome inhibitor MG132 (MG, 10µM)-, or tunicamycin (Tm, 2µg/ml)-treated cells transfected with UiFC-N and UiFC-C. Cells were imaged every 10 min for up to 240 minutes. (B, C) Graphs show changes in the UiFC fluorescence intensities, normalized as a fold increase over the initial intensity (taken as 1 at time 0 min) in untreated cells or cells treated with MG132, tunicamycin, or doxorubicin. Inset in (B) shows changes in UiFC fluorescence every min for 9 min of MG132 treatment. Data in (B and C) represent mean +/-SD (n > 70 in all experimental groups). (D) Co-localization of K48 chains (blue) and UiFC fluorescence (yellow) under ER stress. HeLa cells co-expressing UiFC-N and UiFC-C were treated with tunicamycin treatment for 4 hours as in (A) followed by immunofluorescent staining for K48 chains (blue). (E) Effects of UiFC-N and UiFC-C on ubiquitination *in vitro*. Increasing amounts of 6His-UiFC-N and 6His-UiFC-C was added in the reaction mix contains human E1, Ube2g2, gp78C, ubiquitin and ATP (see details in Experimental Procedures) (ATP+) and incubated for 3 hours followed by measuring UiFC fluorescence on a fluorescent microplate reader. The same reaction mix without ATP (ATP-) or with ATP and EBV-DUB (DUB+) was performed as controls. The graph shows mean +/- SD of triplicate wells. The reaction mixes were analyzed by immunoblotting for ubiquitin after the measurement of UiFC fluorescence. (F) UiFC-N and UiFC-C concentration-dependent effects on ubiquitination *in vitro*. Increasing amount (0, 0.125, 0.25, 0.5, 1, 2, and 4 µg of each) of 6His-UiFC-N and 6His-UiFC-C were included in *in vitro* polyubiquitination mix as described in (E). Ubiquitin chains were analyzed by immunoblotting for ubiquitin.

We noticed that many cells transfected with the UiFC-N and UiFC-C exhibited faint homogeneous venus fluorescence and contained few, if any, concentrated puncta, which probably reflects low levels of basal polyubiquitination that is constantly occurring throughout the cell. If this is the case, we would expect to see increases in fluorescence in cells treated with proteasome inhibitor because of accumulation of polyubiquitinated proteins. Another possibility is that the low fluorescence stems from random re-association of the venus fragments in UiFC-N and UiFC-C, thereby will not be altered by proteasome inhibition. To determine whether proteasome inhibition affects the homogeneous venus fluorescence, HeLa cells co-expressing the UiFC-N and UiFC-C reporters were treated with proteasome inhibitor, MG132, followed by time-lapse imaging of UiFC-reporter fluorescence every 10 minutes for up to 4 hours (Figure 8A, middle panel and Movie S2). In control untreated cells the UiFC fluorescence increased slightly over the 4-hour period (1.50±0.34 fold increase from the initial level, n=74; Figure 8A, upper panel). By contrast, MG132 treatment induced a more robust time-dependent increase in the UiFC fluorescence (5.45±2.04 fold increase over the initial level, n=78; Figure 8A, upper panel). Importantly, a steep increase in the UiFC fluorescence was detected within minutes of MG132 treatment (Figure 8B, inset), indicating that UiFC is able to rapidly report on dynamic increases in K48 polyubiquitin chain in live cells.

Misfolded proteins in the endoplasmic reticulum (ER) are conjugated with K48 or K11 chains and subsequently delivered to the proteasome for degradation by the ER-associated degradation pathway (ERAD) [24,46,47]. We therefore investigated whether UiFC could be used to track changes in polyubiquitination during ERAD, by treating cells with tunicamycin to cause a build-up of misfolded proteins in the ER [48]. Consistent with increasing proteasomal degradation, tunicamycin treatment induced an initial slow increase (~1.1 fold/hour) in UiFC fluorescence for about 30 minutes followed by a faster increase (~2.3 fold/hour) from 60 to 90 minutes, which was followed by a return to the rate of about 1.1 fold/hour (Figure 8C and Movie S3). UiFC fluorescence induced by tunicamycin treatment colocalized well with anti-K48 chain immunostaining (Figure 8D), indicating the ubiquitin-proteasome-dependent degradation is activated at the ER. The importance and the mechanism underlying the dynamics in the changes of polyubiquitination as revealed by UiFC fluorescence following tunicamycin treatment remain to be determined.

Doxorubicin is commonly used anti-cancer drug that triggers apoptosis through a DNA damage response pathway [49,50]. It remains controversial whether doxorubicin increases or decreases polyubiquitination in cells [49,50]. We found that treatment of cells with doxorubicin induced a slow but steady increase in the UiFC fluorescence for up to 4 hours (Figure 8D, Movie S4), indicating the drug induces an increase in polyubiquitination in cells.

A particular concern with any UBD-based ubiquitin chain reporter is that they can potentially interfere with the normal dynamics of ubiquitination. One way that this could be avoided is to ensure expression of the reporters is sufficiently low to alleviate the problem, as typified by the expression levels we used in the figures shown in Figure 7A. To directly determine the dose-dependent effects of UiFC-N and UiFC-C on polyubiquitination we utilized an *in vitro* ubiquitination assay. Recombinant UiFC-N and UiFC-C were added to a ubiquitination reaction mixture containing gp78C (E3), E1, Ube2g2, ubiquitin and ATP as previously reported [36]. Control reactions included omission of ATP from or addition of the deubiquitination enzyme (DUB) to the reaction mixture. Three hours after the reaction, UiFC fluorescence was measured on a fluorescence microplate reader and then the reaction mixtures were examined for polyubiquitin by immunoblotting. The results showed that despite the presence of UiFC-N and UiFC-C, ubiquitination still occurred (Figure 8E). Omission of ATP or using DUB markedly reduced ubiquitination (Figure 8E). Furthermore, ubiquitination was not significantly altered even when the concentration of the UiFC-N and UiFC-C was increased by four-times more than the amount we chose for detecting polyubiquitin chains *in vitro* (Figures 1D and 3 and Figure 8F, 0.86 µM vs. 3.44 µM). These results suggest that this UIM-based UiFC, when used at adequate amounts, do not interfere significantly with polyubiquitination and thus, is suitable for monitoring changes in ubiquitination and deubiquitination *in vitro* and *in vivo*.

## Discussion

Here we describe the development of a UiFC assay for measuring and tracking changes in the dynamics of K48 polyubiquitination *in vitro* and *in vivo*. Several properties distinguish UiFC from other available fluorescent ubiquitin chain sensors [23,32]. First, UiFC is based on the principle of BiFC, whereas the recently reported chains sensors are UBD directly tagged with a fluorescent protein. Therefore, the fluorescence of UiFC is reconstituted by increases in abundance of ubiquitination in cells, whereas the fluorescence of the reported sensors is constantly present, which could mask proper detection of ubiquitination and cannot detect the changes in abundance of ubiquitination in cells. Second, UiFC can quantitatively detect K48 chains *in vitro* with excellent signal response and reproducibility, whereas the reported sensors can only been used in cells. Third, UiFC can be used to monitor changes of diffused ubiquitination but the reported sensors can only be used to localize ubiquitin chains with distinct subcellular localization.

Many E3 ubiquitin ligases and DUBs are potential drug targets [51–53]. UiFC can be easily adapted for *in vitro* high-throughput screening for drugs that target E3s or DUBs, since it requires only a pair of engineered proteins, UiFC-N and UiFC-C, to quantify the abundance of ubiquitin chains. Moreover, for ubiquitin chain quantification, UiFC-N and UiFC-C can be added after ubiquitination reaction or directly included in ubiquitination or deubiquitination reaction mixture. Therefore, UiFC can be truly a one-step screening assay for inhibitors of E3 ubiquitin ligases and DUBs. The flexibility and convenience contrasts with the current *in vitro* ubiquitination techniques that utilize special ubiquitin labels, such as ^32^P or HRP, or use labeled anti-polyubiquitin antibody as readout for high throughput screening [54–56]. Moreover, these assays require washing steps, which is time-consuming and also makes the assays prone to well-to-well variation. Fluorescence resonance energy transfer (FRET) and time-resolved-FRET (TR-FRET)-based screening assays overcome the problem associated with conventional assays but are technically demanding and requires expensive reagents and instrumentation [57,58]. UiFC is simple and cost-effective, and has good signal response and high reproducibility. The Z score of UiFC is considered to be in the “ideal” range [42]. Therefore, UiFC, or modifications of it, could be an excellent alternative for measuring the abundance of ubiquitin chains in high throughput screening for modulators of ubiquitination or deubiquitination by E3 ubiquitin ligases or DUBs.

We have obtained evidence suggesting that the epsin1 UIM-based UiFC preferentially detects K48 ubiquitin chain. First, the UiFC fluorescence was preferentially generated by the presence of K48 ubiquitin chains *in vitro* in a dose-dependent manner, but much less so by the presence of K11 or K63 chains. Second, the UiFC fluorescence colocalizes well with anti-K48 ubiquitin chain antibody staining. Third, inhibition of proteasomal degradation causes a time-dependent increase in UiFC fluorescence. This is consistent with the fact that K48 ubiquitin chains are one of the primary signals targeting proteins for proteasomal degradation [24–26]. Fourth, activation of ERAD induces a time-dependent increase in UiFC fluorescence in cells and decorates a reticular pattern consistent with ubiquitin-dependent degradation of proteins from the ER. Additionally, the reticular UiFC fluorescent signals colocalize well with anti-K48 chain antibody staining. Previous studies have reported that both K11 and K48 chains are proteasome-targeting signals that are used in ERAD [24,46,47]. Interestingly, we showed that UiFC was slightly increased in the presence of K11 chains *in vitro*, and thus the increase in UiFC fluorescence in cells seen during tunicamycin-induced ERAD likely stems chiefly from an increase in K48-linked ubiquitination. Our *in vitro* and *in vivo* assays indicate that UiFC does not significantly respond to the presence of K63-linked ubiquitin chains. The K48 linkage selectivity of UiFC is surprising since the UIMs of epsin1 that we used for construction of UiFC-N and UiFC-C have been shown to interact equally well with K48 and K63 chains *in vitro* [34]. Even more puzzling is that we also found that the UIMs of epsin1 are also capable of binding K11 chains *in vitro*. One possible explanation for the K48 chain selectivity of UiFC is that although the UiFC-N and UiFC-C bind all three types of chains (K11, K48 and K63), the distinct topology of K48 chains position the venus fragments in UiFC-N and UiFC-C in such a way that favors complementation of fluorescence. Obviously, when bound to K11 and K63 ubiquitin chains this complementation is largely unfavorable. Despite the selectivity of UiFC for detecting K48 chains *in vitro*, the existence of ubiquitin chains with heterologous linkages makes it difficult to detect pure K48 chains in cells.

In summary, we show that the epsin1 UIM-based UiFC provides a new tool for studying K48-linked changes in ubiquitination *in vitro* and in live cells. In principle, this UIM-based UiFC could easily be modified for detection and monitoring changes in other ubiquitin and related (e.g. SUMO) chains, provided that the UBD that are utilized allows complementation of fluorescence when binding to the ubiquitin or related chains.

## Methods

### Plasmids and antibodies

To construct pcDNA3-UiFC-N and pcDNA3-UiFC-C, the cDNA (accession # NP_476477.1) encoding rat epsin1 amino acid residues 166-256 fused with the cDNA encoding the N-terminal fragment of the yellow fluorescent protein Venus (V–N: amino acid residues 1-173) or the C-terminal fragment of Venus (V–C: amino acid residues 154-240) was cloned into pcDNA3 vector through NotI and XbaI sites. The amino acid residues 166-256 fragment of rat epsin1 contains the three tandem UIMs [34]. A linker sequence N-IDGGGGSGGGGSSG-C was included between UIMs and V–N or V–C. Venus was split as reported previously [35]. For expression of recombinant 6His-tagged UiFC-N and UiFC-C, DNA fragment encoding either UiFC-N or UiFC-C was cloned into pQE-60 derived vector through the NcoI */*HindIII sites, respectively. To make pQE-60-EBV-DUB, DNA fragment encoding the protease domain (aa1-270) of the Epstein–Barr Virus (EBV) large tegument protein BPLF1 (EBV-DUB) [41] was synthesized by SBI (Canada) and amplified by PCR and then inserted into the NcoI (blunted by Klenow) */HindIII* sites for expression of recombinant 6His-tagged EBV-DUB. Mammalian expression vectors encoding, untagged Parkin, CFP-Parkin, mCherry-Parkin, and p62/SQSTM1 were kind gifts from Drs. Richard J. Youle (NIH, Bethesda, MD, USA) and Terje Johansen (University of Tromsø, Tromsø, Norway), respectively [45,59]. All expression vectors were verified by DNA sequencing prior to use.

The primary antibodies used for immunofluorescence were: rabbit monoclonal anti-K48 ubiquitin chain (clone Apu2) (Millipore), rabbit monoclonal anti-K11 ubiquitin chain (clone 2A3/2E6) (Millipore), rabbit monoclonal anti-K63 ubiquitin chain (clone Apu3) (Millipore) and mouse monoclonal anti-K63 ubiquitin chain antibody (clone HWA4C4) (Millipore), rabbit polyclonal anti-GFP-HRP antibodies (Rockland), mouse monoclonal anti-ubiquitin-HRP antibody (Santa Cruz Biotech), rabbit polyclonal anti-p62/SQSTM1 antibodies (Sigma), mouse monoclonal anti-p62/SQSTM1 antibody and FK2 anti-ubiquitin antibody (Cell Signaling), and rabbit polyclonal anti-Tom20 antibodies (Santa Cruz). Secondary antibodies, including anti-mouse and anti-rabbit IgG antibodies conjugated with Alexa Fluor 350 (blue) or Alexa Fluor 594 (red) were purchased from Invitrogen.

### In vitro ubiquitination

*In vitro* ubiquitination assay was modified from the protocol reported previously [36–38]. To synthesize K11 and K63 chains, the reaction mix containing human E1 (30 nM), E2 (500 nM, Ube2s for K11 and Ubc13/Uve1a for K63), ubiquitin (10 µM) in buffer (25 mM Tris/HCl (pH 7.4), 2 mM ATP, and 2 mM MgCl_2_) were incubated overnight at 37 °C. For synthesis of K48 chain, 4 µM recombinant gp78C were added to the reaction mix and used Ube2g2 as E2. Expression and purification of 6His-tagged gp78C (pET28a-gp78C) was done as reported previously [36]. The same reaction mixes without ATP were prepared as negative controls for K11, K48, or K63 chains. The linkage of the resulted ubiquitin chains was confirmed by immunoblotting with linkage-specific antibody.

### Interaction of ubiquitin chains with UiFC-N and UiFC-C

The interaction was assessed by immunoprecipitation following the protocol published previously [36]. Briefly, 1 µg of purified UiFC-N or UiFC-C were incubated with indicated ubiquitin chains and anti-GFP antibodies in buffer containing 150mM NaCl, 1mM EDTA, 1mM EGTA, 10mM Tris/HCl (pH 7.4), 0.5% NP-40, and protease inhibitors. And then protein A-sepharose beads were added to the reaction to precipitate the immune-complex followed by immunoblotting of the precipitates for UiFC-N, UiFC-C and ubiquitin.

### In vitro UiFC

Expression and purification of 6His-UiFC-N, 6His-UiFC-C, and 6His-EBV-DUB were performed according to the procedure reported previously [60]. For *in vitro* UiFC, 0.84 µM UiFC-N and UiFC-C were added to indicated amount of ubiquitin chains in 20 µl Tris buffer (25 mM, pH 7.4) and time-dependent changes in UiFC fluorescence was measured using excitation at 488 nm and emission at 535 nm at 37 °C every 10 minutes for the indicated time on TECAN Infinite 200Pro multimode microplate reader. Since the ubiquitin chains synthesized were variable in length, it is not possible to calculate its molar concentration. Therefore, all ubiquitin chain concentration indicated in figures were represented using mono-ubiquitin concentration used for synthesizing the chains. To determine the effects of UiFC-N and UiFC-C on ubiquitination *in vitro*, UiFC-N and UiFC-C were included in above described ubiquitination reaction mix and UiFC fluorescence was measured.

The Z factor of *in vitro* UiFC was calculated using the formula reported previously [42].

### In vivo UiFC

HeLa cells were cultured in Dulbecco’s Modified Eagle Medium (DMEM) supplemented with 10% heat-inactivated fetal bovine serum, 2 mM GlutaMAX, 1 mM sodium pyruvate, MEM non-essential amino acids (GIBCO), 100 U/ml penicillin, and 100 mg/ml streptomycin in 5% CO_2_ at 37°C. Plasmids were transfected with indicated plasmids using Lipofectamine2000 (Invitrogen) or Extreme GENE9 DNA transfection reagent (Roche), according to the manufacturers’ instructions. Transfected cells were analyzed within 16-24 hours post transfection. Immunofluorescent staining was done as reported previously [36].

For time-lapse imaging, cells were cultured in 2-well chambered borosilicate coverglasses (Nunc). Prior to imaging phenol red-containing culture medium was replaced with phenol red-free, fully supplemented DMEM. Images were acquired on a motorized stage-equipped Zeiss AxioObserver Z1 fluorescent microscope, using either a 100/1.45 a-Plan-FLUAR objective lens (Zeiss MicroImaging; for Parkin experiments), or 63/1.40 Plan-Apochromat objective lenses (Zeiss MicroImaging, for other studies). Time-lapse experiments were carried out on a Zeiss AxioObserver Z1 microscope at 37°C and 5% CO_2_, conditions set by an environment control unit (Zeiss MicroImaging). Definitive Focus module (Zeiss MicroImaging) was used to prevent focal plane drifts during time-lapse image acquisition. Images were recorded using a high-sensitivity QuantEM 512SC CCD camera (Photometrics). To normalize cell exposure to the light, the setting for the single channel imaging was kept constant throughout all experiments so that acquisition of single image took 5 ms. Image analyses were performed using AxioVision 4 (Zeiss MicroImaging) or ImageJ (NIH) softwares. Graphs and statistical analyses were obtained with Microsoft Excel software.

## Supporting Information

Movie S1 **Time-lapse-imaging of UiFC and mitochondria translocation of mCherry-Parkin in FCCP-treated cells.** Plasmids encoding mCherry-Parkin, UiFC-N and UiFC-C were transfected into HeLa cells. The following day cells were treated with FCCP to induce mitochondrial damage and subsequent mCherry-Parkin translocation to the mitochondria, and analyzed by time-lapse imaging for mCherry-Parkin and UiFC every 5 minutes for up to 70 minutes. Red: mCherry-Parkin; green: UiFC; merge of red and green.
(MOV)

Movie S2 **Time-lapse-imaging of UiFC in MG132-treated HeLa cells.** UiFC-N and UiFC-C-expressing HeLa cells were treated with the proteasome inhibitor MG132 followed by time-lapse imaging of UiFC fluorescence every 10 minutes for up to 4 hrs.
(MOV)

Movie S3 **Time-lapse-imaging of UiFC in tunicamycin-treated cells.** HeLa cells co-expressing UiFC-N and UiFC-C were treated with the N-glycosylation inhibitor tunicamycin followed by time-lapse imaging of UiFC fluorescence every 10 minutes for up to 4 hrs.
(MOV)

Movie S4 **Time-lapse-imaging of UiFC in doxorubicin-treated cells.** HeLa cells co-expressing UiFC-N and UiFC-C were treated with the cancer chemotherapy drug doxorubicin followed by time-lapse imaging of UiFC fluorescence every 10 minutes for up to 4 hrs.
(MOV)
