# Correction: Ubiquitination-Induced Fluorescence Complementation (UiFC) for Detection of K48 Ubiquitin Chains *In Vitro* and in Live Cells

**DOI:** 10.1371/annotation/65881dc3-5bce-4f0b-9060-7079e16b1877

**Published:** 2013-09-23

**Authors:** Zhiliang Chen, Yongwang Zhong, Yang Wang, Shan Xu, Zheng Liu, Ilia V. Baskakov, Mervyn J. Monteiro, Mariusz Karbowski, Yuxian Shen, Shengyun Fang

Affiliation number 1 for all authors besides the ninth author is incorrect. The correct affiliation is: Center for Biomedical Engineering and Technology, University of Maryland, Baltimore, Maryland, United States of America.

In addition, affiliation number 2 for the first, second, third, fifth and last authors is incorrect. The correct affiliation is: Department of Physiology, University of Maryland, Baltimore, Maryland, United States of America.

Finally, the last author is not affiliated with institution number 4, but only institutions numbers 1 and 2. 

